# IMMEDIATE COMPLICATIONS AFTER 88 HEPATECTOMIES - BRAZILIAN CONSECUTIVE SERIES

**DOI:** 10.1590/0102-6720201600030012

**Published:** 2016

**Authors:** Enio Campos AMICO, José Roberto ALVES, Samir Assi JOÃO, Priscila Luana Franco Costa GUIMARÃES, Joafran Alexandre Costa de MEDEIROS, Élio José Silveira da Silva BARRETO

**Affiliations:** University Hospital Onofre Lopes, Federal University of Rio Grande do Norte, Natal, RN, Brazil

**Keywords:** Hepatectomy, Postoperative complications, Morbidity, Mortality

## Abstract

**Background:**

Hepatectomies have been increasingly recommended and performed in Brazil; they present great differences related to immediate complications.

**Aim:**

Assessing the immediate postoperative complications in a series of 88 open liver resections.

**Method:**

Prospective database of patients subjected to consecutive hepatectomies over nine years. The post-hepatectomy complications were categorized according to the Clavien-Dindo classification; complications presenting grade equal to or greater than 3 were considered major complications. Hepatic resections involving three or more resected liver segments were considered major hepatectomies.

**Results:**

Eighty-four patients were subjected to 88 hepatectomies, mostly were minor liver resections (50 cases, 56.8%). Most patients had malignant diseases (63 cases; 71.6%). The mean hospitalization time was 10.9 days (4-43). Overall morbidity and mortality rates were 37.5% and 6.8%, respectively. The two most common immediate general complications were intra-peritoneal collections (12.5%) and pleural effusion (12.5%). Bleeding, biliary fistula and liver failure were identified in 6.8%, 4.5% and 1.1% of the cases, respectively, among the hepatectomy-specific complications.

**Conclusion:**

The patients operated in the second half of the series showed better results, which were apparently influenced by the increased surgical expertise, by the modification of the hepatic parenchyma section method and by the increased organ preservation.

## RESUMO

**Racional:**

No Brasil as hepatectomias têm sido cada vez mais indicadas e realizadas, apresentando grandes diferenças relacionadas às complicações imediatas.

**Objetivo:**

Avaliar as complicações pós-operatórias imediatas em uma série de 88 ressecções hepáticas abertas.

**Método:**

Foi utilizada uma base de dados prospectiva de pacientes submetidos à hepatectomias consecutivas em nove anos. As complicações pós-hepatectomia seguiram a Classificação de Clavien-Dindo, sendo consideradas complicações maiores aquelas as quais apresentaram grau igual ou maior que 3. Foram consideradas hepatectomias maiores as ressecções hepáticas que envolveram três ou mais segmentos hepáticos ressecados.

**Resultados:**

Oitenta e quatro pacientes foram submetidos a 88 hepatectomias, sendo a maioria ressecções hepáticas menores (50 casos; 56,8%). A maior parte dos pacientes apresentou doença maligna (63 casos; 71,6%). O tempo médio de internação foi de 10,9 dias (4-43). A taxa de morbidade e mortalidade global foi, respectivamente, de 37,5% e 6,8%. As duas complicações gerais imediatas mais frequentes foram as coleções intraperitoneais (12,5%) e o derrame pleural (12,5%). Já as complicações específicas das hepatectomias - sangramento, fístula biliar e insuficiência hepática - foram respectivamente de 6,8%, 4,5% e 1,1% dos casos.

**Conclusão:**

Os pacientes operados na segunda metade da presente casuística tiveram melhores resultados influenciados, aparentemente, pela maior experiência cirúrgica, modificação do método de secção do parênquima hepático e maior preservação do fígado.

## INTRODUCTION

Nowadays, liver resections are indicated for a large number of benign and malignant diseases. The great progress made in the last 30 years was responsible for the reduction in surgery-related mortality from 10% (in the 1980s) to less than 4%^12^. There are few studies about the consecutive series of patients operated in the same health service or by the same team of surgeons in Brazil. In addition, the hepatectomy-related complication rates alone range from 0%^26^ to 56.5%^5^, and it may suggest possible methodological biases in such studies. 

The aim of this study was to present the results of post-hepatectomy complications in a consecutive series of patients.

## METHOD

This research was approved by the University Hospital Onofre Lopes Ethics Committee of the Federal University of Rio Grande do Norte, Natal, RN, Brazil. It was based on the prospective database of patients subjected to consecutive liver resections (through laparotomy), which were performed by the main author (ECA), from July 2006 to July 2015, at Onofre Lopes University Hospital, Federal University of Rio Grande do Norte and Casa de Saúde São Lucas Hospital, Natal, RN, Brasil. The following information were collected and analyzed: the clinical features of the patients and of the underlying diseases, the liver resection type, the associated surgical procedures, the minor or major postoperative complications, need of blood transfusion, the hospitalization time, and the mortality up to 90 postoperative days.

The preoperative assessment of the cardiovascular, pulmonary and renal systems, as well as of the patient´s nutritional status, was common and standard in all patients subjected to hepatectomy, with few specific changes related to the underlying disease. Only patients with compensated *cirrhosis* and hepatocellular carcinoma, without portal hypertension (Child A), were taken into consideration for surgical treatment.

There were at least 30 days between the last chemotherapy cycle and the surgical treatment in cases of patients undergoing chemotherapy for colorectal liver metastasis. The preoperative portal embolization was assessed in cases in which a small liver residue was predicted.

Patients with cholangiocarcinoma, supposed to undergo right hepatectomy, were subjected to preoperative external biliary drainage. They were operated just after the total bilirubin values got below 5 mg/dl.

The hepatectomy surgical procedure was standardized. General anesthesia associated with epidural anesthesia was applied to non-cirrhotic patients supposed to undergo major liver resection. A large-caliber venous vascular access (caliber=8.5 Fr) was set in the right internal jugular vein and in the arterial access (left radial artery) in order to measure the mean arterial pressure of all patients during the anesthetic induction. Restricted intravenous hydration was performed during the liver pre-resection stage in order to keep the central venous pressure low (less than 5 mmHg). The Pringle maneuver was selectively used in cases of substantial bleeding in the hepatic parenchyma resection stage. The "Glissonian Approach" was used in the hepatic pedicle ligation, whenever possible. Preference was given to the extrahepatic dissection of the hepatic veins. The Silkclasy technique^10^ was used to section the hepatic parenchyma in the first half of the series (first 44 cases), whereas the ultrasonic aspirator was used in the second half of it (CUSA^(r)^ Excel + Ultrasonic Surgical Aspirator). Metal clips were used in the hemostasis of small blood vessels in the hepatic parenchyma, whenever they were available. Most recently, the linear stapler was used in a few cases (the last 10 cases) to section the Glissonian pedicle or the hepatic veins of the patients. The routine postoperative drainage was performed using silicone laminar drains, except for cases in which minor resections were mainly performed in the left hepatic lobe.

According to the Clavien-Dino classification, the postoperative complications were categorized as major, whenever they presented grade equal to or greater than 3, and as minor, whenever the grade was lower than 3^4^. Cases demanding necessary resection in 3 or more liver segments were considered major hepatectomies^11^.

The diagnosis of post-hepatectomy complications such as bleeding, biliary fistula and liver failure, was made according to the International Study Group for Liver Surgery^13^ ^,^ ^21^ ^,^ ^22^. Thus, the post-hepatectomy bleeding was defined as more than 3 g/dl hemoglobin level decrease at the end of the surgery, in comparison to the preoperative levels; and/or as the need of postoperative erythrocyte transfusion due to hemoglobin decrease, and/or as the invasive re-intervention to control bleeding. The post-hepatectomy liver failure was defined as the hepatic function deterioration characterized by increased INR (international normalized ratio); or as the need of injecting clotting factors to keep the INR normal; or as the occurrence of hyperbilirubinemia from the fifth postoperative day on. The post-hepatectomy biliary fistula was defined as the presence of intra-peritoneal liquid or of liquid from the abdominal drain presenting high total bilirubin levels (at least three times higher than the serum level) from the third postoperative day on; or as the need for surgery or interventional radiology to drain a bile collection; or as the re-operation due to biliary peritonitis.

Mortality was taken into consideration whenever the death occurred within the first 90 postoperative days.

## RESULTS

Eighty-four patients were subjected to 88 hepatectomies. Their mean age was 54.01 years (14-89) and most of them were women (n=55; 62.5%). Six were diagnosed with cirrhosis. Most had malignant diseases (n=63; 71.6%); colorectal metastasis and hepatocellular carcinoma appeared most often (Table 1).

**TABLE 1:** Distribution of diseases that were the basis for the indication of 88 hepatectomies in nine years

Hepatectomy indications	Frequency
Benign diseases	
Hepatic adenoma	6
Biliary cystadenoma	6
Intrahepatic lithiasis	4
Focal nodular hyperplasia	3
Hemangioma	2
Liver trauma	1
Latrogenic bile duct injury	1
Acute calculous cholecystitis	1
Non-specific benign disease	1
Malignant diseases	
Primary:	
Hepatocellular carcinoma	9
Gallbladder adenocarcinoma	7
Cholangiocarcinoma	4
Malignant epithelioid cell tumor	1
Secondary:	
Metastatic colorectal cancer	30
Metastatic neuroendocrine tumor	5
Metastatic breast cancer	3
Metastatic gastrointestinal stromal tumor	1
Metastatic squamous cell carcinoma of the cervix	1
Metastatic squamous cell carcinoma of the anal canal	1
Metastatic gallbladder adenocarcinoma	1
TOTAL	88

Twenty-eight (28) patients (31.81%) were subjected to some surgical procedure associated with hepatic resections; biliodigestive anastomosis (n=6) and colon resection (n=4), respectively, were the most often performed ones.

**FIGURE 1:**
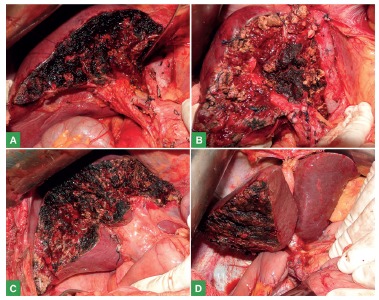
Major hepatectomy illustrative cases in final aspect of liver resections at the end of the intraoperative period: A) left hepatectomy + caudate resection + lymphadenectomy of the hepatic hilum due to intrahepatic cholangiocarcinoma; B) left hepatectomy + caudate resection due to metastatic colon adenocarcinoma with highlighting that the resection extends to the Glissonian pedicle of the right hemi-liver in order to obtain a negative margin; C) resection of liver segments II + III + IVb + V due to metastatic colon adenocarcinoma; D) right hepatectomy + caudate resection + lymphadenectomy + resection of the biliary tract and Roux-en-Y reconstruction due to cholangiocarcinoma in the confluence of the hepatic ducts (Klatskin Tumor).

**FIGURE 2:**
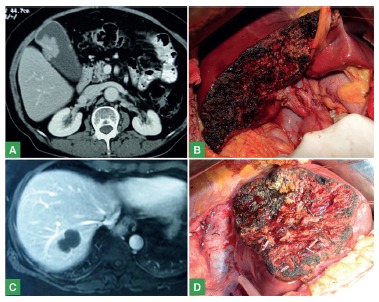
Minor hepatectomy illustrative cases: A) abdomen tomography showing gallbladder wall vegetation through enhanced intravenously administered contrast; B) final aspect of IVb + V bisegmentectomy + lymphadenectomy of the hepatic hilum with anatomopathological examination resulting in gallbladder adenocarcinoma; C) abdominal tomography showing hypodense lesion in segment VII topography with involvement of the right hepatic vein in patient with history of invasive ductal breast carcinoma; D) final appearance of liver segment VII resection associated with resection of the right hepatic vein with anatomopathological examination resulting in metastatic adenocarcinoma

Fifty (56.8%) minor and 38 (43.2%) major hepatectomies were performed (Table 2, Figures 1 and 2).

**TABLE 2:** Distribution of the 88 hepatectomies performed in nine years, according to liver resection type

Hepatectomy types	Frequency
(Resected liver segments)	
Minor hepatectomies	
* Monosegmentectomies:	
Seg II	1
Seg III	3
Seg IV	2
Seg IVb	2
Seg V	3
Seg VI	2
Seg VII	2
Enucleation	2
* Bisegmentectomies:	
Seg II and III	14
Seg IV and VIII	1
Seg IVb and V	7
Seg V and VI	5
Seg VI and VII	3
Seg VII and VIII	3
Major hepatectomies	
Right hepatectomy	9
Right hepatectomy + caudate	2
Right trisegmentectomy	1
Mesohepatectomy	2
Left hepatectomy + caudate	4
Left hepatectomy	14
Resection of segments II, III, IVb and V	1
Resection of segments II, III and VII	2
Resection of segments II, VI and VII	1
Resection of segments IVb, VII and VIII	1
Resection of segments V, VI and VIII	1
TOTAL	88

Seg=resection of liver segment(s); + =associated with resection

Twenty-seven (27) hepatectomies (30.7%) required blood transfusion (2.3 units of red blood cell concentrate per patient on average; ranging from 1 to 8 blood bags). Twenty-three (23) out of these cases required up to 2 blood bags. The patients remained 2.4 days (0-17), on average, in the intensive care unit, and the mean postoperative hospital stay was 10.9 days (4-43). They were divided according to the hepatic parenchyma dieresis technique and those who were operated in the second half of the series (the last 44 cases in the series), using the CUSA^(r)^ type scalpel, showed better results (Table 3).

**TABLE 3:** Comparison between results of the first half (the 1st 44 hepatectomies) and the second half (the last 44 hepatectomies) in patients operated in nine years

Sample features	First half	Second half
(n=88)	(n=44)	(n=44)
Age (mean)	55.2 years	52.4 years
Malignant/benign disease ratio	32 / 12	31 / 13
Major/minor hepatectomy ratio	21 / 23	17 / 27
Right/left resection ratio	14 / 22	15 / 18
Mean hospital stay	12.7 days	9.1 days
Number (%) of patients who received [ ] red blood cell transfusion	15 (34%)	12 (27.3%)
Mean number of [ ] red blood cell bags per patient	3 blood bags	1.75 blood bags
Mean postoperative ICU time	2.9 days	1.8 days
Incidence of complications (overall)	43.20%	31.80%
Complications directly related to the hepatectomies:		
- Incidence of intra-abdominal collection	15.9%	9.1%
- Incidence of postoperative bleeding	11.4%	2.3%
- Incidence of biliary fistula	2.3%	6.8%
- Incidence of liver failure	2.3%	0.0%
Incidence of major complications	20.40%	9.10%
Incidence of deaths	11.30%	2.30%

n=number of hepatectomies performed; [ ]=concentrate; %=percentage of cases; ICU= intensive care unit

**TABLE 4:** Distribution of postoperative complications after 88 hepatectomies

Postoperative	Frequency
complications	n (%)
Intraperitoneal collection	11 (12.5%)
Pleural effusion	11 (12.5%)
Bleeding	6 (6.8%)
Surgical wound infection	5 (5.7%)
Biliary fistula	4 (4.5%)
Incisional hernia	4 (4.4%)
Pneumonia	3 (3.4%)
Urinary tract infection	2 (2.3%)
Pleural empyema	2 (2.3%)
Atelectasis	2 (2.3%)
Acute myocardial infarction	1 (1.1%)
Liver failure	1 (1.1%)
Portal vein thrombosis	1 (1.1%)
Acute intestinal obstruction	1 (1.1%)
Liver abscess	1 (1.1%)
Pulmonary failure	1 (1.1%)
Pulmonary thromboembolism	1 (1.1%)
Pneumothorax	1 (1.1%)
Ascites	1 (1.1%)
Central catheter infection	1 (1.1%)

n=occurred cases; %=percentage

The overall morbidity and mortality rates were, respectively, 37.5% and 6.8%. On the other hand, the morbidity and mortality rates related just to patients subjected to major resections were, respectively, 47.3% and 10.5%, and those related to patients subjected to minor resections were, respectively, 30% and 4.0%.

## DISCUSSION

The first successful elective liver resection for liver tumor removal was performed in Berlin by Carl Von Langenbuch in 1887^16^. The first hepatectomy reports in Brazil date back to the 1950s^7^. However, liver resections have become routine in surgical practice only in the last 30 years. It happened due to several advances in the medical knowledge, such as the development and improvement of complementary imaging tests, the dissemination of safe surgical and anesthetic techniques, the better postoperative care, and the improvement in knowledge about liver regeneration^8^. Nowadays, these advances, all together, allow performing hepatectomies with minimal morbidity and mortality rates, which is crucial to expand the hepatectomy indications, mainly in oncology and in liver transplant fields^8^.

Although the hepatectomy-related morbidity and mortality rates are not the only ones, they are an important efficiency marker of these procedures. Thus, the incorporation of a standardized system to identify such events is desired. The Clavien-Dindo classification has been progressively accepted since its inception in 2004^4^. In addition, more recent studies have validated such classification for several surgery types^2^ ^,^ ^27^ ^,^ ^29^. This research used this classification to categorize the overall postoperative complications, whereas the specific postoperative complications (liver failure, bleeding and biliary fistula) were categorized through definitions suggested by the International Study Group for Liver Surgery^13^ ^,^ ^21^ ^,^ ^22^. Therefore, the overall postoperative complication, the major complication and the mortality rates found in the current study were 37.5%, 14.7% and 6.8%, respectively. Although the herein found complication rate is within the world average, the death rate was higher than that described in the recent national literature, i.e., it was between 1-5%^25^. The death rate in the second half of the series was similar to the best national and even to the international levels, only after the sample was divided in two halves. In addition, it was almost five times lower (11.3% vs. 2.3%) than the casuistic in the first half of the series. It is believed that there were three possible main reasons for the improved results. Firstly, the second half of the sample showed greater tendency to minor hepatectomies and followed the trend in the literature of saving the maximum amount of liver parenchyma as possible^15^. Secondly, there was less need of transfusion, less perioperative bleeding and less incidence of postoperative intraperitoneal collections, probably due to the ultrasonic surgical aspirator used to section the parenchyma in the second half of the series. Last, but not least, the team of surgeons and anesthetists experimented a learning curve related to the selection and monitoring of patients for surgery, besides the standardization of surgical procedures.

The major complications in this series were those with high lethality and others with less death potential. Three patients were identified in the first group; each of them presented one of the following complications: acute myocardial infarction, postoperative liver failure and thrombosis of the mesenteric-portal axis. All three patients died. Based on such cases, the patients presenting risk of coronary disease in the preoperative period were investigated based on higher suspicion index. In addition, the hepatic volume calculation was extended to patients subjected to right hepatectomy as a way to reduce the risk of postoperative liver failure. No factor able to be corrected through the management of patients was identified in the prevention of portal vein thrombosis, because the attachment of the falciform ligament to the abdominal wall was already used as routine practice in the right hepatectomy to avoid portal vein kinking^14^. The second group comprised only patients who developed bleeding, abdominal collection and biliary fistula, which led to death incidence of 33%, 33% and 25%, respectively. The diagnosis of complication was easier in these patients and it was obtained through clinical assessment and laboratory analysis of the liquid collected from the abdominal drain. In addition, the most effective treatment comprised blood transfusions and drainage of intra-abdominal abscesses or of the biliary leakage.

Liver resections are performed at several reference services throughout Brazil; however, there are few studies showing the overall immediate results of such procedure. In most cases, such studies comprise few patients, sometimes with specific diseases or even subjected to specific types of liver resections. Often, the immediate complications are not reported or, in case they are reported, they are not evenly defined. The literature review resulted in 14 studies about patients subjected to at least 40 liver resections in Brazilian medical services in the past 20 years ^3^ ^,^ ^5^ ^,^ ^6^ ^,^ ^9^ ^,^ ^10^ ^,^ ^15^ ^,^ ^17^ ^-^ ^20^ ^,^ ^23^ ^,^ ^24^ ^,^ ^26^. 

Disregarding the study by Fernandes ^6^, because it comprises a series of patients subjected to hepatectomy for donation in living donor liver transplants, most case series comprised patients with non-cirrhotic liver (84.3 - 100%) and with malignant disease (48-96.6%), fact that meets the most prevalent type of patient in the current study. The number of major hepatectomies in the other national case series was quite varying (from 9.6-61.4%). The complications were descriptively reported in 12 studies, whereas only one^5^ included the Clavin-Dindo Classification in its methodology. Such fact certainly influenced the big difference evidenced in the complication rates, i.e., a range from 0-56.6%. It is almost certain that the minor complications categorized as grade I were not taken into consideration in the results of the studies that did not use the Clavin-Dindo Classification, due to their small impact.

The only Brazilian study that could be compared to the herein presented series has retrospectively analyzed 129 resections in non-cirrhotic livers, 42.6% of these series were considered major hepatectomies^5^. The morbidity and mortality rates were 56.5% and 7.8%, respectively, whereas those of the current study were 37.5% and 6.8%, respectively. The mortality rate was very similar in both studies; however, the complication in the aforementioned study was higher than the herein described. The complication types also showed some differences: 1) this study found similar prevalence of pulmonary and infectious complications (both with 20.5%), whereas Feier et al.^5^ found pulmonary and infectious complications in 39.7% and 28.7%, respectively; 2) this research found biliary fistula index three times lower (4.5x13.7%) than that reported by Feier et al.^5^; and 3) this paper found bleeding rate almost three times higher (6.8x2.7%) than that found in the aforementioned study.

The biliary fistula did not represent a major problem in the herein investigated series. The methylene blue test was systematically performed after the liver resections, despite the lack of validated studies to prove its eficiency^25^. Diluted dye (1:3 parts of saline solution) was injected in the cystic duct during the test in order to identify its presence in the open wound of the resected liver. Whenever dye leakage was detected, a suture was performed by applying separate "X" stitches using Prolene 4-0^(r)^. It is believed that the herein described test helped preventing postoperative biliary fistulas. The treatment in all four biliary fistula cases was conservatively conducted and based on the maintenance of the abdominal drains left at the time of surgery.

## CONCLUSION

Patients operated in the second half of the series showed better results, which were apparently influenced by the increased surgical expertise, by the modification in the hepatic parenchyma section method, and by the increased organ preservation.
